# Contrasting strategies to cope with drought conditions by two tropical forage C_4_ grasses

**DOI:** 10.1093/aobpla/plv107

**Published:** 2015-09-02

**Authors:** Juan Andrés Cardoso, Marcela Pineda, Juan de la Cruz Jiménez, Manuel Fernando Vergara, Idupulapati M. Rao

**Affiliations:** 1 Tropical Forages Program, International Center for Tropical Agriculture (CIAT), Apartado Aéreo 6713, Cali, Colombia; 2 Facultad de Ciencias Agrarias, Universidad Nacional de Colombia, Carrera 30 No. 45-03, Bogotá, Colombia

**Keywords:** Anisohydric–isohydric behaviour, biomass allocation, projected shoot areas, root distribution, soil water content, stomatal density

## Abstract

Drought severely limits forage productivity of C_4_ grasses across the tropics. The avoidance of water deficit by increasing the capacity for water uptake or by controlling water loss are common responses in forage C_4_ grasses. Napier grass (*Pennisetum purpureum*) and *Brachiaria* hybrid cv. Mulato II are tropical C_4_ grasses used for livestock production due to their reputed resistance to drought conditions. However, there is scant information on the mechanisms used by these grasses to overcome water-limited conditions. Therefore, assessments of cumulative transpired water, shoot growth, leaf rolling, leaf gas exchange, dry mass production and a number of morpho-physiological traits were recorded over a period of 21 days under well-watered or drought conditions. Drought reduced shoot dry mass of both grasses by 35 %, yet each grass exhibited contrasting strategies to cope with water shortage. Napier grass transpired most available water by the end of the drought treatment, whereas a significant amount of water was still available for Mulato II. Napier grass maintained carbon assimilation until the soil was fairly dry, whereas Mulato II restricted water loss by early stomatal closure at relatively wet soil conditions. Our results suggest that Napier grass exhibits a ‘water-spending’ behaviour that might be targeted to areas with intermittent drought stress, whereas Mulato II displays a ‘water-saving’ nature that could be directed to areas with longer dry periods.

## Abstract (precis)

The study provided an overview of the dynamics of growth, water uptake and water use of two tropical C_4_ forage grasses: Napier grass (*Pennisetum purpureum*) and *Brachiaria* hybrid cv. Mulato II. This in combination with the observations of leaf rolling scores suggested that Napier grass and Mulato fall respectively into contrasting “*water spending*/*water saving*” models of water use. As such, Napier grass might be targeted for areas with intermittent and short periods of drought, whereas Mulato II might be a better option for areas with longer drought spells.

## Introduction

Grass-dominated ecosystems occupy ∼33 % of Earths' vegetative area (Shantz 1954; Jacobs *et al*. 1999) and provide most of the forage to feed domestic livestock (Morgan *et al*. 2011). Across the tropics, cultivated grasses used to sustain livestock are mostly C_4_ perennials of African origin (Sarmiento 1992; Williams and Baruch 2000; Peters *et al*. 2013). Grasses showing the C_4_ photosynthetic pathway often show greater competitive ability than C_3_ species under dry and high irradiance environments (e.g. tropical grasslands and savannas) (Pearcy and Ehleringer 1984; Edwards *et al*. 2010; Taylor *et al*. 2011, 2014). This competitive advantage is brought up by the ability of C_4_ species to maintain greater photosynthetic rates per unit of water loss than C_3_ species (Sage and Kubien 2003; Taylor *et al*. 2014). However, water availability is still critical in determining the productivity of C_4_ grasses and wide variability has been found in how they respond to periods of drought (Ludlow 1976; Wedin 2004).

The development of water deficit in C_4_ grasses, as in any plant, occurs when the water supply does not match the water needs. Leaf rolling is a common response in C_4_ grasses under such conditions and has been widely used as an indicator of stress/resistance to water limitation (e.g. *Andropogon halli*, *Sporobolus cryptandrus*, Phillips Hardy *et al*. 1995; *Cenchrus* species, Amaresh and Dubey 2009; *Zea mays*, Blum 2011; *Sorghum bicolor*, Neilson *et al*. 2015). The perennial nature of cultivated tropical forage grasses means that these plants must face water-limiting periods at some point or another (Ludlow 1980). As such, the avoidance of water deficit either by increasing the capacity of water uptake or by controlling water loss are common responses in tropical C_4_ grasses (Williams and Baruch 2000). Deep root systems, with greater root length densities with increasing soil depth, have been generally linked with uptake of stored water in lower layers of soil (Baruch and Fernández 1993; Guenni *et al*. 2004; Zhou *et al*. 2013, 2014). Extensive root systems maximize water extraction, minimizing the reduction of leaf transpiration (*E*) that might result from growth under drying soil. On the other hand, control of plant water loss has been related to the restriction of *E* (Sarmiento 1992; Zhou *et al*. 2012; Cathey *et al*. 2013). The amount of *E* is in turn influenced by leaf area, leaf stomatal density and mainly regulated by stomatal opening/closure (Lawson and Blatt 2014), usually estimated by stomatal conductance (*g*). Although key in regulating water loss, stomata also exert control over CO_2_ entry needed for photosynthesis (Lawson and Blatt 2014). Hence, reductions of *E* by stomatal closure can be reflected in lower CO_2_ assimilation rates (*A*), and thereby and inevitably, in reduced growth.

A fine interplay exists between the acquisition of water by roots in drying soil and water loss through transpiration. These two components tend to act simultaneously (Blum 2011). Nonetheless, several authors have shown behaviours of how plants cope with water-limiting conditions. Some plants showed transpiration levels under drying soil similar to those of well-watered plants (Hund *et al*. 2009; Lopes and Reynolds 2010; Henry *et al*. 2011). In contrast, others exhibited a decline of *E*, even in relatively wet soil (Kholová *et al*. 2010*a*; Zaman-Allah *et al*. 2011; Ries *et al*. 2012). These behaviours are commonly associated with the anisohydric (‘water-spending’) and isohydric (‘water-saving’) model of water use. The pertinence of each model of water use depends on the pattern of drought events that plants might have to deal with (Kholová *et al*. 2010*a*). The anisohydric model might be relevant to maintain plant growth, without major yield penalties, under periods of short and/or intermittent drought. Conversely, the isohydric model of water use might be more relevant to maintain growth, albeit reduced, under extended periods of drought (Kholová *et al*. 2010*a*, *b*; Ries *et al*. 2012; Vadez *et al*. 2013).

Over 600 million people of low income depend on livestock production to sustain their livelihoods (Perry and Sones 2007; Herrero *et al*. 2009). Most of them live in the developing tropics (Thornton *et al*. 2002). Since cultivated tropical C_4_ grasses are primarily rain-fed (Herrero *et al*. 2012), it is imperative to define forage options that fit current and future precipitation scenarios. Forage C_4_ grasses widely planted in tropical regions include Napier grass (*Pennisetum purpureum*; Mwendia *et al*. 2013) and *Brachiaria* hybrid cv. Mulato II (Pizarro *et al*. 2013). One of the factors behind the wide adoption of these two grasses is their reputed forage productivity under drought conditions. To our knowledge, most of this information comes from either grey literature or anecdotal accounts. Although important, these sources of information provide incomplete information of mostly preliminary results. This partially explains conflicting information of the productivity and resilience of Napier grass and Mulato II to limited water supply within one agro-ecological zone or across different agro-ecological zones. Moreover, studies separating forage productivity from coping mechanisms to drought conditions in these two grasses are largely absent.

The aim of this work was therefore to improve the understanding of the responses and coping strategies of two tropical forage C_4_ grasses (Napier grass and Mulato II) under limited water supply conditions. This information could contribute to the targeting of Napier grass and Mulato II to specific agro-ecological zones with certain patterns of drought events. Hence, measurements of leaf gas exchange (*A*, *g* and *E*), non-destructive estimations of shoot growth and assessment of leaf rolling were performed in conjunction with the determination of gravimetric soil water content throughout 21 days of plant growth under well-watered and drought conditions. To determine sites of water extraction, the volumetric water content down the soil profile was determined at the end of the trial. Leaf area, leaf stomata density, number of roots, root length density (RLD), average root diameter (ARD) and dry mass of shoots and roots were recorded before and at the end of the experimental period. To determine patterns of biomass accumulation, the ratio of root dry mass to shoot dry mass was calculated at 0 and 21 days of treatment. Additionally, the ratio of root length to foliar area was calculated before and at the end of the trial.

## Methods

### Plant material and growing conditions

Napier grass (*P. purpureum*) and *Brachiaria* hybrid cv. Mulato II are perennial C_4_, warm-season grasses. Napier grass is native to Eastern and Central Africa, whereas Mulato II is a hybrid of three *Brachiaria* grasses (*B. decumbens*× *B. brizantha*× *B. ruziziensis*). The genotypes used in this study corresponded to the following accession numbers of the International Center for Tropical Agriculture (CIAT) forage gene bank (Napier grass: 26850; cv. Mulato II: 36087). Napier grass grows sparse and tall erect culms up to 4.5 m whereas cv. Mulato II grows a dense tussock up to 1.3 m height (Peters *et al*. 2011).

Establishment, growing conditions and harvesting of plants were similar to that previously described in Beebe *et al*. (2013) and Cardoso *et al*. (2014). The trial was conducted in a greenhouse at CIAT headquarters, Palmira, Colombia (latitude 3°29′N; longitude 76°21′W; altitude 965 m). During the course of the experiment, atmospheric conditions were recorded at a weather station (WatchDog 2475 Plant Growth Station, Spectrum Technologies Inc., USA) and run at an average temperature of 29/21 °C (day/night), a relative humidity of 45/70 % (day/night) and a maximum photosynthetic active radiation (PAR) of 1050 µmol m^2^ s^−1^ (average daily PAR value of 710 µmol m^2^ s^−1^). Soil used in this study was an Oxisol collected from Santander de Quilichao, Department of Cauca in Colombia (latitude 3°60′N; longitude 76°310′W; altitude 990 m) at 0–0.20 m from the soil surface. Soil was sieved to pass a mesh of 0.002 m.

Plant material used in this study consisted of vegetative propagules of both grasses that were grown in pots filled with 4 kg of fertilized soil (milligrams of nutrient added per kilogram of soil: N 21, P 26, K 52, Ca 56, Mg 15, S 10, Zn 1.0, Cu 1.0, B 0.05 and Mo 0.05) and well-watered conditions. Each propagule was visually selected for homogeneity (∼0.04 m length); and had a differentiated node for rooting and a single expanding leaf. Propagules were then re-planted in a 2 : 1 (w/w) mixture of soil and river sand that was previously fertilized (milligrams of nutrient added per kilogram of soil mixture: N 40, P 50, K 100, Ca 101, Mg 28, S 20, Zn 2.0, Cu 2.0, B 1.0 and Mo 1.0). This rate of fertilization represented the recommended fertility level for crop-pasture establishment in tropical acid soils (Rao *et al*. 1992). After fertilization, soil mixture was allowed to air dry for a couple of days. Soil mixture presented a bulk density (*ρ* soil) of 1400 kg m^−3^, organic matter of 4 % and a pH of 4.4. After the soil was air-dried, 5 kg of soil mixture was loaded in transparent plastic cylinders (0.8 m high × 0.075 m diameter) inserted in beige polyvinyl chloride pipes of the same dimensions. The soil mixture was then saturated with water and allowed to drain for a couple of hours until reaching field capacity. After that, one propagule was planted ∼0.01 m below the soil surface in each soil cylinder and watered daily to maintain field capacity under greenhouse conditions. Due to differences in the rate of establishment of the two grasses, Mulato II propagules were allowed to grow for 21 days, and those of Napier grass were grown for 14 days before the start of the experiment. The potted plants were randomly organized in the greenhouse. For each grass, 4 out of 22 propagules that were initially planted were discarded based on poor growth and wilting. An initial harvest of four randomly selected plants per grass was done to have baseline data for shoot and root dry mass, leaf area, leaf stomatal density, number of roots, root length, ARD and RLD (methodology described below). After that, a factorial combination of two genotypes (Napier grass and Mulato II) by two water supply conditions (well-watered and progressive drying of soil) in a seven-replicate complete randomized block was established, and the experiment was conducted for 21 days. Previous work identified an evaluation period (∼21 days of establishment plus 21 days of treatment) as adequate to minimize the impact of container size (0.8 m high × 0.075 m diameter) on root growth and as to avoid ratios of total plant dry masses to pot volume larger than 2 kg m^−3^ (Poorter *et al*. 2012). Furthermore, the use of such containers proved effective to minimize the effect of a perched water table (<0.01 m) at the bottom of the cylinders.

### Cumulative transpired water

The amount of evapotranspired water was monitored by weighing each cylinder throughout the experiment every 2 days and prior to harvesting (at 21 days). Soil of well-watered treatment was maintained at field capacity by the addition of the same mass of water lost through evapotranspiration in a 2-day period. The progressive drying of soil treatment (from now on referred as drought) was imposed by cessation of watering from the start of the experiment. Soil cylinders without plants were used to estimate evaporation of soil alone under the two levels of water supply. Cumulative transpired water was calculated according to Cabrera-Bosquet *et al*. (2009) from the difference between evapotranspiration and evaporation (average value of seven cylinders).

### Assessment of shoot growth and leaf rolling

Changes in shoot area and leaf rolling throughout the experiment were estimated from digital images. A preliminary trial indicated that calculation of projected shoot areas (PSAs) using two perpendicular front views of Napier grass and Mulato II (at 0 and 90°) yield equivalent results to shoot areas estimated using three views of plants (one top view and two perpendicular front views), which in turn were positively correlated with leaf area determined destructively (*r* = 0.9, *P*< 0.01). For that reason and simplicity, only two front views of each plant (0 and 90°) were acquired. Digital images were captured at 0, 7, 14 and 21 days from the onset of the experiment with a stationary digital camera (Coolpix p6000, Nikon Corporation, Japan). Images were acquired in two time slots during the day, from 9:00 to 9:30 h (pre-noon) and from 11:30 to 12:00 h (noon). Images taken at pre-noon were used to estimate PSA. Field of view of digital images corresponded to a black and mat background of 1.96 × 1.47 m. Projected shoot areas was estimated by transforming colour images into binary ones (black and white) using ImageJ software (v. 1.38, National Institutes of Health, USA) and by counting white pixels (i.e. PSA in image) out of the black background (i.e. field of view). Untransformed images taken at pre-noon and noon were used for the visual assessment of leaf rolling at 0, 7, 14 and 21 days from the onset of the experiment following the categories described by Engelbrecht *et al*. (2007). Briefly, six leaf rolling scores in plants were used as follows: 1 (no leaf rolling); 2 (some leaf rolling); 3 (severe leaf rolling; slightly wilted); 4 (severely wilted; necrosis in leaf tips); 5 (nearly dead); and 6 (dead). Since changes in evaporative demand during the day might affect expression of leaf rolling, vapour pressure deficits at pre-noon and noon were recorded and obtained from the greenhouse weather station.

### Leaf gas exchange

The youngest fully expanded and unshaded leaf of every plant was used to monitor the carbon assimilation rate (*A*), stomatal conductance (*g*) and transpiration rate (*E*) at 0, 7, 14 and 21 days after the start of treatments using an infrared gas analyser (Li-COR 6400-XT, Li-COR Biosciences, USA). Measurements were taken between 09:30 and 11:30 h and in the absence of shadowing by clouds. When taking measurements, the leaf chamber of the infrared gas analyser was set up to match ambient conditions of light (∼800 µmol m^2^ s^−1^ of PAR). The leaf chamber was maintained with a concentration of 400 µmol of carbon dioxide and a relative humidity of 50/75 %.

### Stomatal density

Prior to harvesting, colourless nail polish was spread on the adaxial surface of leaves for an imprint of stomata in an area of ∼6 cm^2^ of a leaf. A preliminary trial showed no differences between stomatal density in adaxial or abaxial leaf surfaces of Napier grass or Mulato II. The stomata imprints were viewed under a light microscope (Leitz Ortholux II, Ernst Leitz GmbH, Germany) connected to a digital camera (Coolpix p6000, Nikon Corporation, Japan). Stomatal density (number of stomata per square millimetre) was posteriorly estimated from the recorded images and from the same leaves and areas used for leaf gas exchange measurements.

### Harvest

Plants were harvested 21 days after the start of treatments. Leaves and stems were manually separated, and leaf area was measured with a leaf area meter (Li-COR 3100, Li-COR Biosciences). The leaves and shoots were then oven dried for 96 h at 60 °C for determination of shoot dry mass.

### Morphological characteristics of the root system

Preliminary studies found that root length distribution of Napier grass and Mulato II grown in soil cylinders (0.8 m high × 0.075 m diameter) yielded proportional results to that of plants grown under containers with larger diameters (0.20 m) or grown under field conditions. Furthermore, it was found that root length in both grasses was relatively evenly distributed between 0.10 and 0.60 m from the soil surface under well-watered or drought conditions. Thus, and in the present study, soil cylinders were sliced into five layers representing different depths from the soil surface (0–0.1, 0.1–0.35, 0.35–0.6, 0.6–0.7 and 0.7–0.8 m). Roots were washed free of soil with tap water and then placed in a container with few drops of wetting agent (polysorbate 20) for 10 min and rinsed again with tap water to remove loosened soil. After that, roots of each soil profile were placed into plastic bags and stored at −20 °C for posterior analysis.

For the morphological characterization, roots from each soil depth were carefully placed in an acrylic tray filled with water to minimize the overlapping of roots. Dead roots and debris were removed as much as possible from the tray with tweezers and an eyedropper. Roots were then scanned to record grey images at 400 dpi with a dual scanner (EPSON Expression 1680, Japan). Recorded images were processed with ImageJ software to remove background noise (i.e. soil particles of <0.3 mm). Processed images were then used to estimate root length and ARD using WinRhizo software (Regent Instruments, Canada). The number of nodal roots (main roots developed from the crown) was manually counted from the recorded images. Root length density (the length of roots per unit volume of soil, RLD m m^−3^) at different soil depths was also calculated. After scanning, roots were carefully collected to minimize loss of material and oven dried as described above to determine root dry mass.

### Volumetric determination of soil moisture down the soil profile

A preliminary trial determined that field capacity of the soil mixture used in this study corresponded to 0.33 m^3^ m^−3^ of volumetric water content, and values of 0.13 m^3^ m^−3^ corresponded to the wilting point of plants. Prior to harvesting and washing of roots, a sample of ∼0.05 kg for each soil profile was collected to calculate moisture down the soil profile. Soil samples were weighed immediately after collection (wet soil, kg) and after drying at 105 °C for 48 h (dry soil, kg). Estimation of volumetric soil moisture (*θ*_v_) was calculated using the following formula:

$$\theta_{v}=\left(\frac{\text{wet}\;\text{soil}-\text{dry}\;\text{soil}}{\text{wet}\;\text{soil}}\right)\times \rho \;\text{soil}\;\left(\text{kg}\;{\text{m}}^{-3}\right)\;\times \;\text{density}\;\text{of}\;\text{water}\;\left(\text{kg}\;{\text{m}}^{-3}\right)$$

### Root-to-shoot ratios

The ratio of root dry mass to shoot dry mass (R/S) and the ratio of total root length to leaf area (RL/LA) were calculated on a container basis and for each grass genotype before the start of the experiment and after 21 days of growth under well-watered or drought conditions.

### Statistical analysis

All statistical analyses were performed in R software (v 2.15.2) (R Development Core Team 2012). Data were checked using the Levene's test for homogeneity of variance and log-transformed if necessary. Data were then analysed using repeated-measures ANOVA. A *post hoc* analysis using the Tukey's HSD test (*α*=0.05) was used to identify differences between genotypes, treatments and sampling dates for all the variables tested. Regression analyses for the variables PSA and cumulative transpired water for each genotype and watering treatment were performed. Differences between slopes and intercepts for each pair of the resulting regressions were analysed using the *t*-test.

## Results

### Assessment of cumulative transpired water, shoot growth and leaf rolling

**Figure 1.**
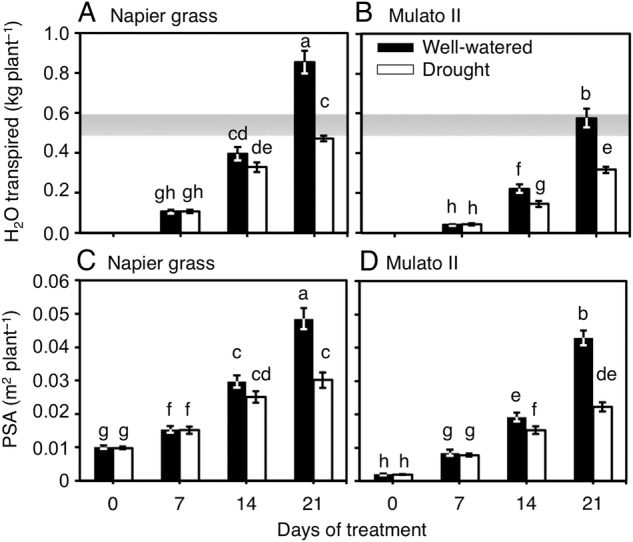
(A) Cumulative transpired water of Napier grass and (B) that of Mulato II grown under well-watered or drought conditions over a period of 21 days. (C) Projected shoot areas of Napier grass and (D) those of Mulato II grown under well-watered or drought conditions over a period of 21 days. Columns represent means and error bars their standard error (*n* = 7). Different letters above columns represent statistical differences at *α* = 0.05 of log-transformed data. The Shaded area represents the total amount of evaporated water under drought conditions at the end of the evaluation period.

**Figure 2.**
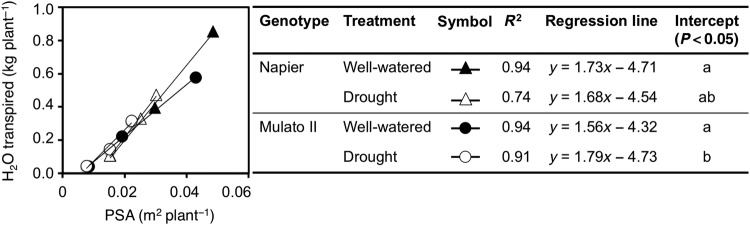
Relationship between PSA and cumulative transpired water of Napier grass and Mulato II grown under well-watered or drought conditions. Symbols represent means of seven replicates. Regression analyses were performed on log-transformed data.

Differences were found among genotypes, watering treatment and sampling dates for cumulative transpired water (Fig. 1A and B; *F*_(11,72)_ = 83.7, *P* = 0.0000, *η*^2^ = 0.9) and PSA (Fig. 1C and D; *F*_(15, 96)_ = 82.7, *P* = 0.0000, *η*^2^ = 0.9). Napier grass showed greater amounts of transpired water and larger PSA throughout the experiment than Mulato II irrespective of watering treatment. Cumulative transpired water and PSA were significantly lower at 21 days of growth under drought conditions for Napier grass (*P*< 0.05, Fig. 1A and C) and from 14 days for Mulato II (*P*< 0.05, Fig. 1B and D). For both grasses and watering treatment, the correlation coefficient (*r*) values for PSA and cumulative transpired water were above 0.85 (*P* < 0.0000) **[see Supporting Information—Table S1]**. The resulting regression lines for each grass and watering treatment were similar and without significant differences between their slopes (Fig. 2) **[see Supporting Information—Table S1]**. However, the intercept of the regression line for Mulato II under drought conditions was significantly different than those of the rest of the regression lines (*P*< 0.05, Fig. 2).

**Figure 3.**
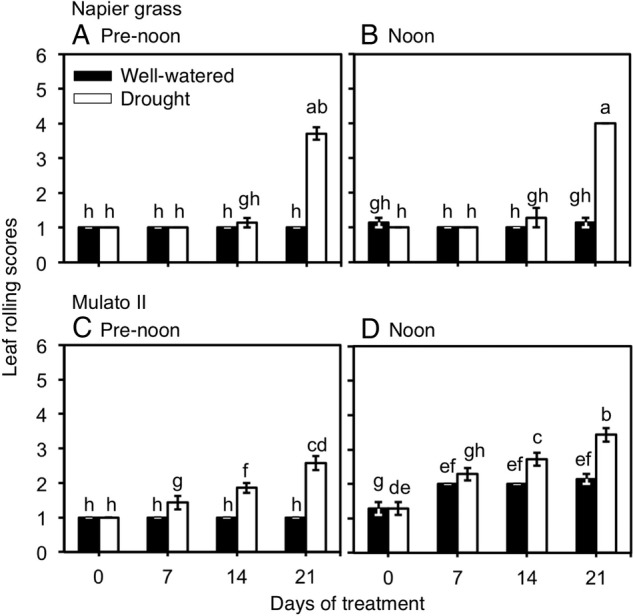
Leaf rolling scores of Napier grass (A and B) and Mulato II (C and D) grown under well-watered or drought conditions over a period of 21 days. Assessment of leaf rolling was performed at pre-noon (lower atmospheric water demand; vapour pressure deficit of ∼1.6 kPa) and noon (higher atmospheric water demand; vapour pressure deficit of ∼5.5 kPa). Columns represent means and error bars their standard error (*n* = 7). Different letters above columns represent statistical differences at *α* = 0.05.

Variation for wilting scores were found among genotypes, watering treatment and sampling dates (Fig. 3A–D; *F*_(31,192)_ = 49.6, *P* = 0.0000, *η*^2^ = 0.9). Napier grass showed a steep increase of leaf rolling scores (up to 4, with starting symptoms of necrosis in the leaf tips) at 21 days under drought conditions (*P*<0.05, Fig. 3A and B). The degree of leaf rolling increased towards noon irrespective of treatment in Mulato (*P*< 0.05, Fig. 3C and D). A gradual increase of leaf rolling scores (up to 3) was noticed for Mulato II under drought conditions (*P*< 0.05, Fig. 3C and D).

### Leaf gas exchange

**Figure 4.**
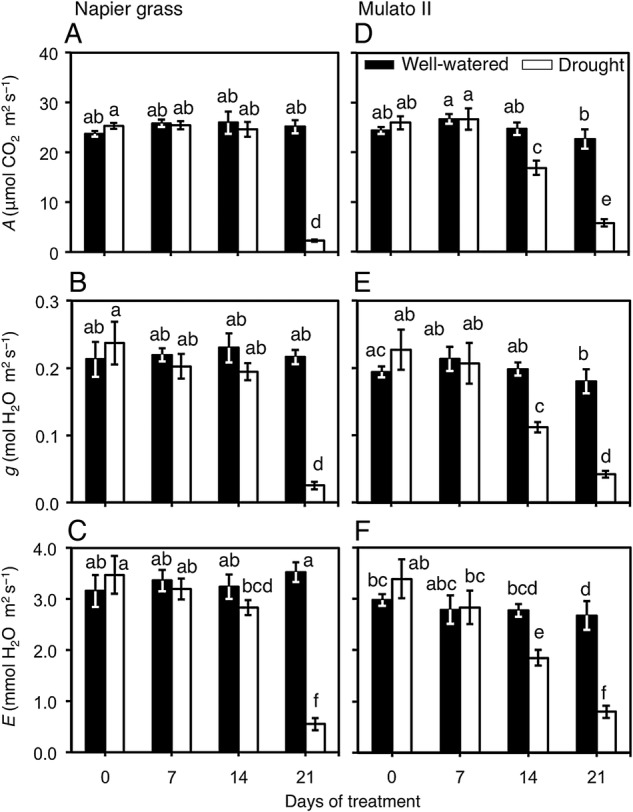
Leaf gas exchange of Napier grass (A–C) and Mulato II (D–F) grown under well-watered or drought conditions over a period of 21 days. *A*, net carbon assimilation rate; *g*, stomatal conductance; *E*, transpiration. Columns represent means and error bars their standard error (*n* = 7). Different letters above columns represent statistical differences at *α* = 0.05.

Variation for leaf gas exchange were found among genotypes, watering treatment and sampling dates: carbon assimilation rate (*A*) (Fig. 4A and D; *F*_(15, 96)_ = 34.7, *P* = 0.0000, *η*^2^ = 0.8); stomatal conductance (*g*) (Fig. 4B and E; *F*_(15, 96)_ = 11.8, *P* = 0.000, *η*^2^ = 0.7); transpiration rate (*E*) (Fig. 4C and F; *F*_(15, 96)_ = 13.9, *P* = 0.000, *η*^2^ = 0.7). After 21 days of drought treatment, Napier grass showed steep reductions of *A*, *g* and *E* (*P*< 0.05, Fig. 4A–C). Reductions of *A*, *g* and *E* were evident for Mulato II after 14 days of growth under drought conditions (*P*< 0.05, Fig. 4D–F).

### Stomatal density

Stomatal density for both grasses before the start of experiment was ∼66 stoma mm^−2^. Compared with baseline data, Mulato II showed a ∼1.4-fold increase in its stomatal density after 21 of growth under well-watered or drought conditions (*P*< 0.05, Table 1). Growth under drought conditions for 21 days did not result in changes for stomatal density for either grass when compared with plants grown for 21 days under well-watered conditions (*P*< 0.05, Table 1).

**Table 1.** Leaf stomatal density of Napier grass and Mulato II before the start of the experiment and after 21 days of growth under well-watered or drought conditions. Data shown are means ± SE (*n* = 4 before experiment; *n* = 7 after 21 days of treatment). Different letters represent statistical differences at *α* = 0.05 [*F*_(5, 30)_ = 18.5, *P* = 0.000], *η*^2^ = 0.8.

	Stomatal density (number of stomata per mm^2^)		
	Before treatment	21 days of treatment	
		Well watered	Drought
Napier grass	66.7 ± 2.3a	74.7 ± 4.4a	65.9 ± 4.1a
Mulato II	66.8 ± 6.0a	93.9 ± 4.7b	100.4 ± 3.9b

### Dry mass production

**Figure 5.**
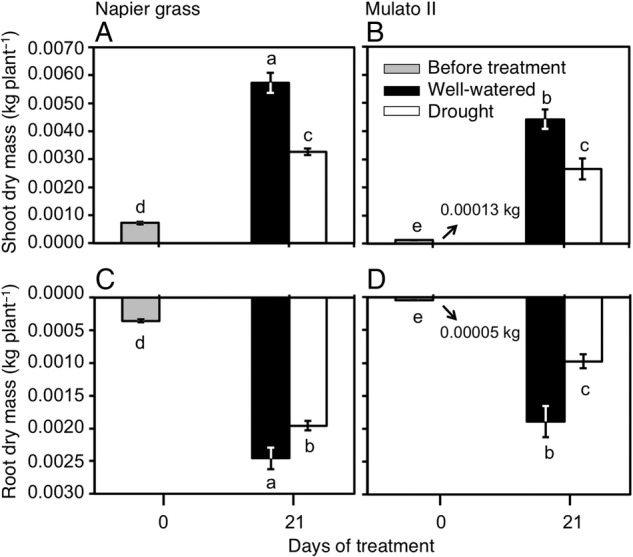
Shoot dry mass of (A) Napier grass and (B) Mulato II; root dry mass of (C) Napier grass and (D) Mulato II before the start of the experiment and after 21 days of growth under well-watered or drought conditions. Columns represent means and error bars their standard error (*n* = 4 before experiment; *n* = 7 after 21 days of treatment). Different letters above columns represent statistical differences at *α* = 0.05 of log-transformed data.

Differences were found among genotypes, watering treatment and harvest dates for shoot (Fig. 5A and B; *F*_(5, 30)_ = 211.1, *P* = 0.0000, *η*^2^ = 1.0) and root dry mass (Fig. 5C and D; *F*_(5, 30)_ = 434.0, *P* = 0.0000, *η*^2^ = 1.0). Prior to the beginning of the experiment, Napier grass showed greater dry masses than Mulato II (*P*< 0.05, Fig. 5). At this point, shoot dry mass was ∼5.6-fold greater and root dry mass was ∼7-fold greater in Napier grass than in Mulato II (Fig. 5). At the end of the experimental period (21 days), shoot and root dry mass of Napier grass were ∼1.5-fold greater than Mulato II under well-watered conditions (*P*< 0.05, Fig. 5). Shoot and root dry masses of Napier grass and Mulato II were significantly reduced under drought conditions (*P*< 0.05, Fig. 5). After 21 days of growth under drought conditions, shoot dry masses for Napier grass and Mulato II were similar and showed a ∼35 % of reduction when compared with their well-watered counterparts (Fig. 5). Under drought conditions, root dry mass was more reduced in Mulato II (50 % reduction) than in Napier grass (20 % reduction).

### Morphology of the root system

**Figure 6.**
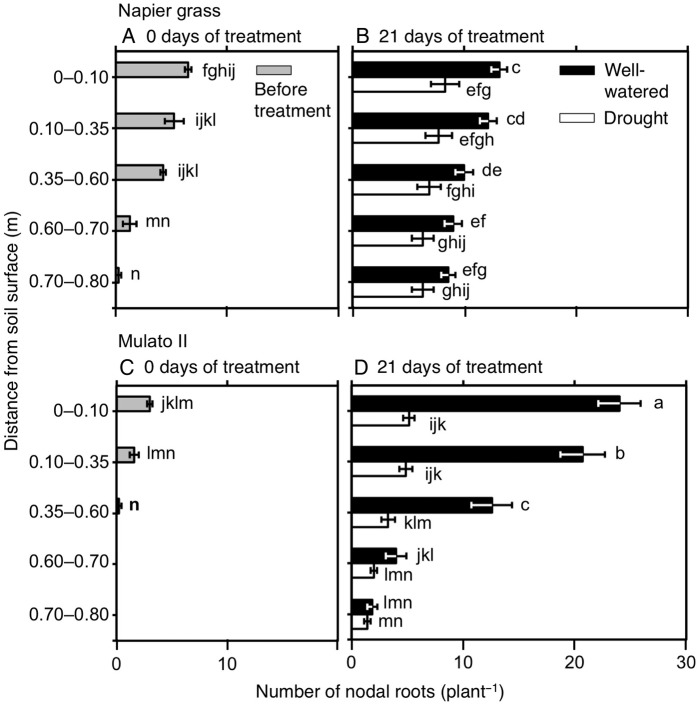
Number of nodal roots down the soil profile of Napier grass (A and B) and Mulato II (C and D) before the start of the experiment and after 21 days of growth under well-watered or drought conditions. Columns represent means and error bars their standard error (*n* = 4 before experiment; *n* = 7 after 21 days of treatment). Different letters above columns represent statistical differences at *α* = 0.05 of log-transformed data.

**Figure 7.**
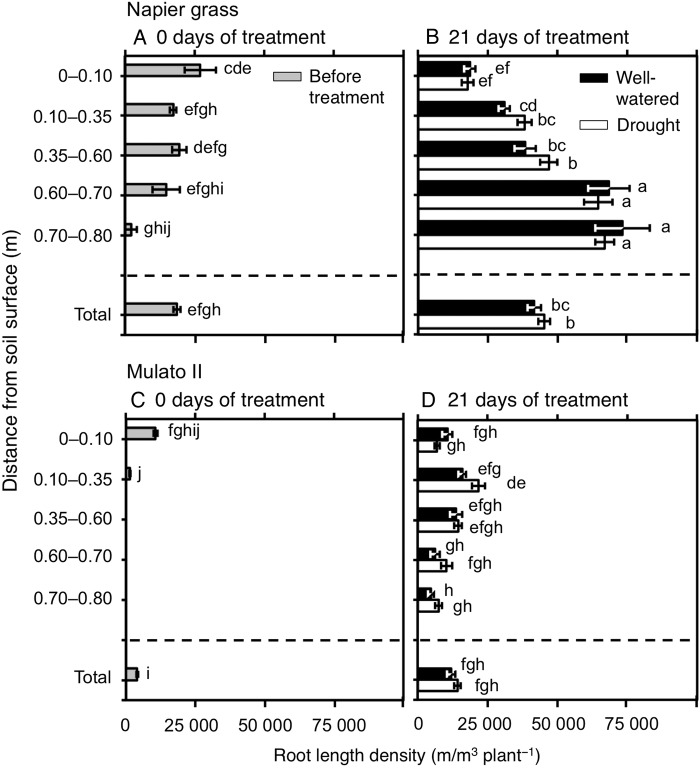
Root length density down the soil profile of Napier grass (A and B) and Mulato II (C and D) before the start of the experiment and after 21 days of growth under well-watered or drought conditions. Columns represent means and error bars their standard error (*n* = 4 before experiment; *n* = 7 after 21 days of treatment). Different letters above columns represent statistical differences at *α* = 0.05 of log-transformed data.

**Figure 8.**
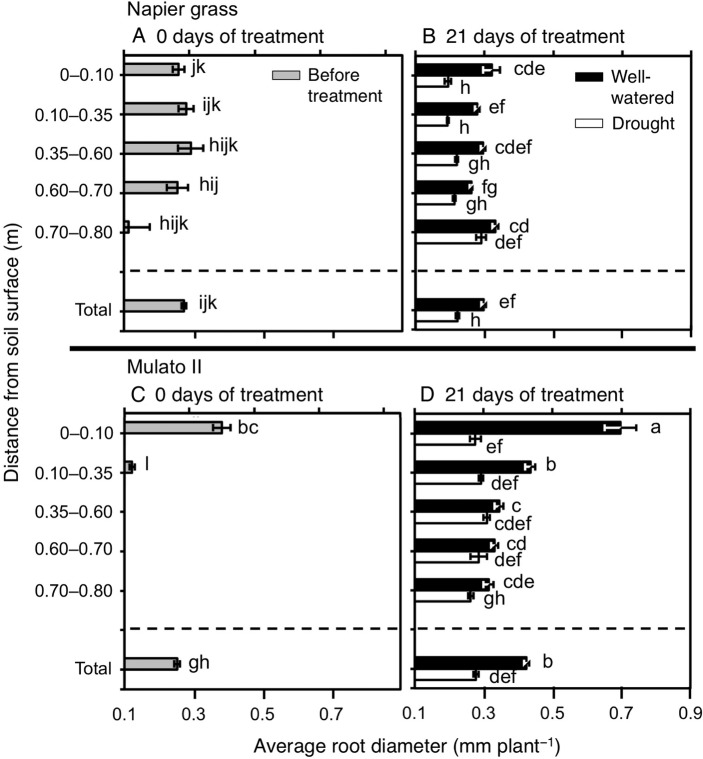
Average root diameter down the soil profile of (A and B) Napier grass and (C and D) Mulato II before the start of the experiment and after 21 days of growth under well-watered or drought conditions. Columns represent means and error bars their standard error (*n* = 4 before experiment; *n* = 7 after 21 days of treatment). Different letters above columns represent statistical differences at *α* = 0.05 of log-transformed data.

The number of nodal roots presented differences among genotypes, watering treatment, distance from soil surface and harvest dates (Fig. 6A–D; *F*_(29, 150)_ = 40.7, *P* = 0.0000, *η*^2^ = 0.9). The number of nodal roots down the soil profile was greater in Napier grass than in Mulato II at the start of the experiment (*P*< 0.05, Fig. 6A and C). Compared with well-watered plants, the number of nodal roots of both Napier grass and Mulato II was significantly smaller after 21 days of growth under drought conditions (*P*< 0.05), but the reduction was more pronounced for Mulato II (Fig. 6B and D). After 21 days of growth under well-watered or drought conditions, most of the nodal roots of Mulato II were located in the 0–0.35 m depth from the soil surface (Fig. 6D), whereas Napier showed a relatively even distribution of nodal roots across the soil profile (Fig. 6B). Variation for RLD was found among genotypes, watering treatment, distance from soil surface and harvest dates (Fig. 7A–D; *F*_(35, 180)_ = 40.5, *P*= 0.000, *η*^2^ = 0.9). At the start of the experiment, RLD of Napier grass was ∼4.5-fold greater than Mulato II (*P*< 0.05, Fig. 7A and C). At the end of the experimental period, total RLD of Napier grass was 3–4 times greater than Mulato II irrespective of treatment (*P*< 0.05, Fig. 7B and D). Total RLD was not affected by drought treatment for either Napier grass or Mulato II (*P*< 0.05, Fig. 7B and D). RLD increased with depth in Napier grass, whereas in Mulato II remained relatively unchanged across soil depth (Fig. 7B and D). The average diameter of the root system (ARD) showed variation among genotypes, watering treatment, distance from soil surface and harvest date (Fig. 8A–D; *F*_(35, 180)_ = 41.7, *P*= 0.000, *η*^2^ = 0.9). Prior to the start of the experiment, ARD of the entire root system of both grasses was similar (∼0.25 mm) (Fig. 8A and C). After 21 days of growth under drought conditions, there was an overall reduction of the ARD for both grasses when compared with well-watered plants (*P*< 0.05, Fig. 8B and D).

### Volumetric determination of soil moisture down the soil profile

**Figure 9.**
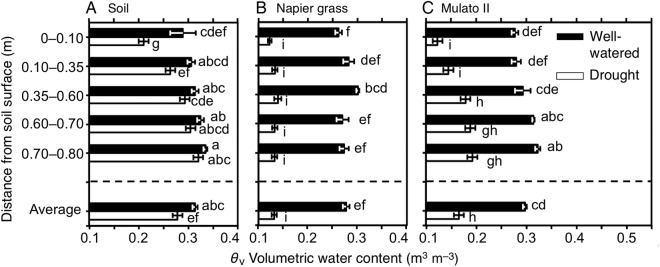
Volumetric water content (m^3^ m^−3^) down the soil profile of (A) soil without plants, and with the presence of (B) Napier grass and (C) Mulato II after 21 days of growth under well-watered or drought conditions. Different letters in front of columns represent statistical differences at *α* = 0.05 of log-transformed data. Volumetric water content of 0.13 m^3^ m^−3^ represents the wilting point of plants, 0.33 m^3^ m^−3^ that of field capacity.

The volumetric water content of soil varied in the presence of different genotypes, watering treatment and distance from the soil surface (Fig. 9A–C; *F*_(35, 216)_ = 87.1, *P*= 0.000, *η*^2^ = 0.9). At the end of the experiment, contents of soil moisture in cylinders without plants and under drying soil were greater with increasing depth (Fig. 9A). Overall, the pattern of soil moisture in the presence of Napier grass was relatively evenly distributed down the soil profile (Fig. 9B). Soil moisture increased with depth in the presence of Mulato II irrespective of water supply treatment (Fig. 9C). At the end of the drought treatment, the volumetric water content of soil (*θ*_v_) where Napier grass plants were grown reached the wilting point of plants (0.13 m^3^ m^−3^). After 21 days of drought and in the presence of Mulato II, moisture conditions were still present from 0.35 m below the soil surface (*θ*_v_ > 0.18) (Fig. 9C).

### Root-to-shoot ratios

**Figure 10.**
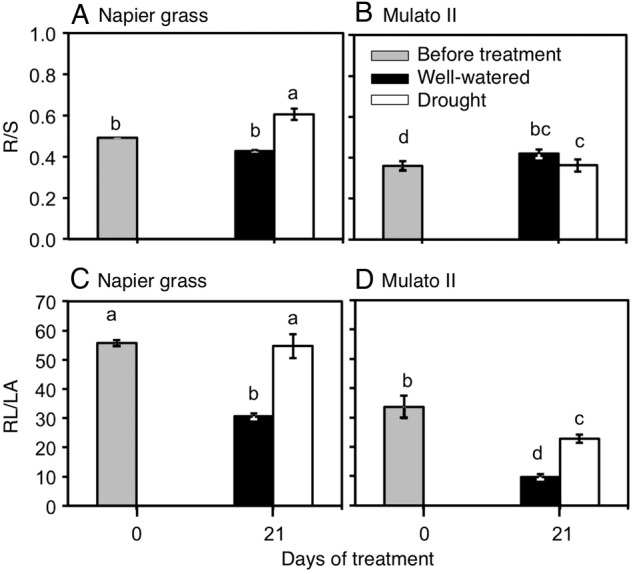
Ratios of root dry mass to shoot dry mass (R/S) (A and B) and root length to foliar area (RL/LA) (C and D) of Napier grass and Mulato II before the start of the experiment and after 21 days of growth under well-watered or drought conditions. Columns represent means and error bars their standard error (*n* = 4 before experiment; *n* = 7 after 21 days of treatment). Different letters above columns represent statistical differences at *α* = 0.05 of log-transformed data.

Variation was found among genotypes, watering treatment and harvest dates for root-to-shoot ratio (Fig. 10A and B; *F*_(5, 30)_ = 64.1, *P* = 0.000, *η*^2^ = 0.7) and the ratio of root length to leaf area (Fig. 10C and D; *F*_(5, 30)_ = 12.8, *P* = 0.000, *η*^2^ = 0.9). The root-to-shoot ratio (R/S) was greater in Napier grass than in Mulato II before the start of the experiment (*P*< 0.05, Fig. 10A and B). Under drought conditions, R/S significantly increased for Napier grass (*P*< 0.05, Fig. 10A). The ratio of root length to leaf area (RL/LA) was greater for Napier grass than Mulato II before the start of the experiment (*P*< 0.05, Fig. 10C–D). Drought treatment increased RL/LA in both grasses but it was significantly larger in Napier grass than in Mulato II (∼2-fold) (*P*< 0.05, Fig 10C and D).

## Discussion

Our results showed that two tropical forage C_4_ grasses (Napier grass and Mulato II) were similarly affected by drought as shown by similar shoot dry masses and a ∼35 % reduction after 21 days of growth under such conditions (*P*< 0.05, Fig. 5A and B). However, the response mechanisms behind such shoot reductions were contrastingly different for each grass. Regression analyses showed that cumulative transpired water was directly proportional to PSA irrespective of genotype or watering treatment (i.e. similar slopes in regression lines, Fig. 2). Yet the magnitude of the regression line (i.e. different intercept in regression lines, Fig. 2) was only significantly different to Mulato II under drought conditions (*P*< 0.05). This difference was probably brought up by tighter stomatal control of leaf gas exchange of Mulato II, relative to Napier grass, under drought conditions. This remark is supported with the observations that time-course reductions of PSA and cumulative transpired water (Fig. 1) coincided with reductions over time of *A*, *g* and *E* under drought conditions (Fig. 4A–F) in both grasses. This indicates that reductions in shoot growth—and the concomitant reduction of cumulative transpired water—were associated with restrictions of leaf gas exchange under drought conditions in these two grasses. Similar responses were previously recorded in Napier grass (Mwendia *et al*. 2013) and other *Brachiaria* forage grasses (Guenni *et al*. 2004).

Stomatal density of Napier grass and Mulato II was similar to densities found in other C_4_ grasses (e.g. *Andropogon gerardii*, Ocheltree *et al*. 2012; 16 grass species, Taylor *et al*. 2012) and it was not affected by growth under drought conditions (Table 1). This suggests that leaf gas exchange, and thereby water loss and growth under drought conditions, is mainly regulated by stomatal control rather than by the reduction of stomatal density in both grasses. Furthermore, since stomata density was ∼1.4-fold greater in Mulato II than in Napier grass at the end of the experimental period (*P*< 0.05, Table 1), yet, values of leaf gas exchange were similar in unstressed plants (Fig. 4A–F), it is likely that stomata size in leaves of Napier grass were larger than in Mulato II.

Carbon assimilation rates (*A*) were sustained for longer under drought conditions in Napier grass than in Mulato II (*P*< 0.05, Fig. 3A and B). This probably contributed to the lesser reduction of root dry mass in Napier grass (20 % reduction) when compared with Mulato II (50 % reduction). Furthermore, the number of nodal roots of both grasses prior to the start of the experiment was slightly less than those after 21 days of drought treatment (Fig. 6A–D). This indicated that for both grasses and under drought conditions, photosynthates were diverted to sustain the growth of existing roots prior to the imposition of the stress, rather than to be used on the development of new ones. Since (i) upper layers of soil dry out before lower layers as drought progresses and (ii) root elongation is highly inhibited by drought (Bengough *et al*. 2010), deeper nodal roots of Napier grass at the onset of the experiment (Fig. 6A) could have facilitated their elongation in the vicinity of more available water with increasing soil depth in drying soil. As such, and together with an inherent small number of nodal roots in Napier grass under well-watered conditions (Fig. 6B), explain the lesser reduction of root numbers (and thereby root dry mass) in Napier grass than in Mulato II under drought conditions.

Root length density was not affected by drought in either Napier grass or Mulato II (Fig. 7B and D). Since the number of nodal roots was greatly reduced by water limitation in both grasses (Fig. 6B and D), the conservative pattern of RLD under drought was either a consequence of an increment of lateral root elongation and/or lateral root production. Increment of lateral root elongation and/or production under drought conditions was implied from the overall reduction of ARD under drought (Fig. 8B and D). Reduction of ARD is considered as an indication of increment of lateral root elongation and/or lateral root production from main roots (Postma *et al*. 2014). The likely increment of lateral root elongation and/or production in these two grasses might in turn have compensated the decline of root surface area brought up by a reduction of nodal root numbers with increasing soil depth under drought conditions.

Napier grass and Mulato II showed RLD above the estimated RLD value of 10 000–15 000 m m^−3^ required to extract most available water in drying soil (Passioura 1983; Wasson *et al*. 2012). Therefore, under drought conditions, Napier grass and Mulato II displayed root systems hypothetically large enough to extract the water resource. Since evaporation of water mainly occurred in the first 0.10 m from soil surface (Fig. 9A), larger RLD than 10 000 m m^−3^ in both grasses in the upper layers of soil at the onset of the experiment (Fig. 7A and C) was most likely beneficial for minimizing loss of water due to direct evaporation of the upper layers of soil in the early phases of the drought stress. The posterior increase of RLD in Napier grass, particularly with increasing depth, far exceeded the value of 10 000–15 000 m m^−3^ (Fig. 7B) that is required to extract most available soil water in drying soil. The anterior coincides with the very low soil moisture content across the soil profile in the presence of Napier grass by the end of the drought treatment (Fig. 9B). Meanwhile, Mulato II only showed slighter RLD value of above 15 000 m m^−3^ in the profile of 0.10–0.35 m from the soil surface at the end of the drought treatment (Fig. 7D), where levels of soil moisture were indeed close to the level of wilting point of plants (Fig. 9C). From 0.35 m below the soil surface, higher levels of soil moisture (Fig. 9C) matched lower values of RLD than 15 000 m m^−3^ in Mulato II under drought conditions. As such, in the presence of Mulato II, low water content in the upper layers of soil was likely to be the outcome of both root water uptake and evaporation, but below 0.35 m of the soil profile, mainly reflected the size of the root system in Mulato II to extract water under drought conditions.

Larger root-to-shoot ratios (R/S) in Napier grass than in Mulato II at early stages and under drought (Fig. 10A and B), suggest that Napier grass invests more assimilates in root growth than Mulato II at these phases and conditions. Increased resource allocation to root growth is thought to increase water acquisition and therefore adaptation to drought (Chimungu *et al*. 2014). However, increased root growth under drought might provide little advantage if a larger shoot, that uses more water is also present (Palta *et al*. 2011; Vadez *et al*. 2013). The potential for water uptake vs. water loss is functionally described by the root length-to-leaf area ratio (RL/LA) (Comas *et al*. 2013). Therefore, the ∼2-fold increase of RL/LA in both grasses under drought conditions (Fig. 10C and D) suggested a phenotypic plasticity to match both the supply and demand of water under drying soil. However, it was noteworthy that Napier grass exhibited a larger RL/LA than Mulato II before and at the end of the experiment. A larger RL/LA together with greater RLD from early in the experiment in Napier grass suggests that Napier grass could be better adapted to acquire and spend the water resource than Mulato II.

Leaf rolling is a common symptom of stress under drought conditions and is an expression of leaf turgor and plant water status (Blum 2011). Stomatal opening/closure respond to both evaporative demand (usually higher at noon) and soil water content, leading to changes in leaf turgor (Martínez-Vilalta *et al*. 2014). Delayed leaf rolling under drought conditions in Napier grass (Fig. 3A and B) could be associated with greater access to water resulting from a larger root system than Mulato II. Yet, the patterns of wilting scores observed throughout the experiment at pre-noon and noon (irrespective of treatment) also suggested a less restricted stomatal control to changes in environmental conditions in Napier grass than in Mulato II. Mulato II, even under well-watered conditions, showed an increase of leaf rolling towards noon (Fig. 3C and D). Restricting transpiration even under non-limiting water conditions in Mulato II most likely contributed to its smaller dry mass production relative to Napier grass under such conditions. Similar observations have been reported with different C_4_ grasses (e.g. *Eragostris spectabilis* vs. *Miscanthus sinensis,* Alvarez *et al*. 2007; 10 C_4_ grasses, Ocheltree *et al*. 2014).

The dynamics of growth, root to shoot ratios, water uptake and water use in combination with the observations of leaf rolling suggest that Napier grass and Mulato II fall, respectively, into the dichotomous anisohydric (‘water-spending’)/isohydric (‘water-saving’) model of water use in plants (c.f. Tardieu and Simonneau 1998; Maseda and Fernández 2006; Sade *et al*. 2012). As such, Napier grass exhibited earlier vigour and a more extensive root system that progressively augmented its size with increasing soil depth. Although larger shoot areas of Napier grass resulted in earlier depletion of soil moisture under drought conditions, the balance of root-to-shoot development leaned towards the growth of roots under drought conditions. A larger root system of Napier grass, in combination with a less restrictive stomatal regulation to limited water supply, resulted in increased carbon assimilation (concomitantly reflected in growth) until the soil became fairly dry. According to this, Napier grass could be an optimal grass for forage production, without big yield penalties, in tropical agro-ecosystems with short, frequent and mild droughts, or where water is available at the bottom of deep soils. Essentially, Napier grass would most likely out-compete Mulato II, in terms of growth, under those prospects of water availability.

On the other hand, Mulato II showed an increase of RL/LA, and values of RLD hypothetically large enough to extract most available water in drying soil. Yet, Mulato II showed reduced transpiration from earlier phases of the experiment under drought conditions indicating stomatal control of water loss rather than the lack of a root system large enough to extract water from the soil. Although earlier stomatal control of transpiration resulted in earlier reductions of shoot growth, saved up water in soil might be used to sustain dry mass accumulation in Mulato II for longer drought periods. In this sense, Mulato II might be better suited than Napier grass for tropical agro-ecosystems where long drought spells are common and a constant supply of forage, albeit reduced, is needed to sustain livestock production.

## Conclusions

This study showed that Napier grass and Mulato II were similar in drought resistance (in terms of the absolute shoot dry mass production and the relative reduction of shoot dry mass over a period of 21 days). However, each grass showed contrasting strategies to cope with water deficit conditions. Napier grass exhibited a larger root system than Mulato II and attempted to maximize carbon assimilation while there was availability of soil water. Conversely, Mulato II showed a root system hypothetically large enough to extract most water under drying soil, yet it restricted water loss by early stomatal closure. This resulted in a faster depletion of water for Napier grass, whereas a significant amount of water was still available for Mulato II at the end of the drought treatment. The aforementioned in combination with observations of leaf rolling indicated that Napier grass showed a ‘water-spending’ behaviour that might be targeted to areas with intermittent drought stress, whereas Mulato II showed a ‘water-saving’ behaviour that could be directed to areas with longer dry periods. Since expression of ‘water-spending’ or ‘water-saving’ behaviours results from the interaction of hormonal regulation and plant hydraulics, we consider that such measurements need to be evaluated in future studies. Further assessments for the ability and rapidness to recover after drought, together with field-based studies, should improve the understanding of responses to limited water supply, but also to improve management practices under such conditions in these two forage C_4_ grasses.

## Sources of Funding

This work was supported by a Swedish International Development Agency funded program (Innovative programmatic approach to climate change in support of BecA's mission: Climate-smart *Brachiaria* grasses for improving livestock production in East Africa).

## Contributions by the Authors

J.A.C. was involved in designing the experiments, data collection and analysis, manuscript preparation and submission. M.P. set up the experiment, collected and analysed data and co-prepared manuscript. J.C.J. contributed to the design, set up of experiment and data analysis. M.F.V. provided technical assistance and collected data throughout the experiment. I.M.R. was involved in project design, supervision of experiments and manuscript preparation.

## Conflict of Interest Statement

None declared.

## Supporting Information

The following additional information is available in the online version of this article –

**Table S1.** Regression analyses for the variables PSA and cumulative transpired water per genotype and watering treatment. Differences between slopes and intercepts for each pair of the resulting regressions were analysed on log-transformed data using the *t*-test (*P* < 0.05).

Additional Information
