# Supplementary material for: Contrasting strategies to cope with drought conditions by two tropical forage C4 grasses
Source: AoB Plants. 2015 Sep 2;7:plv107. doi: 10.1093/aobpla/plv107 (PMC4595746; doi:10.1093/aobpla/plv107)
Supplement: Additional Information [file supp_7_plv107_index.html]

Contrasting strategies to cope with drought conditions by two tropical forage C4 grasses — Contrasting strategies to cope with drought conditions by two tropical forage C4 grasses — Additional Information 

# Contrasting strategies to cope with drought conditions by two tropical forage C4 grasses

## Additional Information

Additional Information

- Additional Information - Docx file
