# Supplementary material for: Contrasting strategies to cope with drought conditions by two tropical forage C4 grasses
Source: AoB Plants. 2015 Sep 2;7:plv107. doi: 10.1093/aobpla/plv107 (PMC4595746; doi:10.1093/aobpla/plv107)
Supplement: Additional Information [file supp_plv107_plv107supp.docx]

**SUPPORTING INFORMATION**

**File 1. Table. Regression analyses for the variables PSA and cumulative transpired water per genotype and watering treatment. Differences between slopes and intercepts for each pair of the resulting regressions were analysed on log transformed data using the *t* test (*p* < 0.05).**

| Genotype | Napier | Napier | Mulato | Mulato II | Napier | Mulato II |
| --- | --- | --- | --- | --- | --- | --- |
| Treatment | Well-watered | Drought | Well-watered | Drought | Well-watered | Well-watered |
| n | 21 | 21 | 21 | 21 | 21 | 21 |
| *r* | 0.9738 | 0.86 | 0.972 | 0.953 | 0.9738 | 0.972 |
| Slope |  |  |  |  |  |  |
| *t* | -0.2238 | | 1.4404 | | -1.2466 | |
| df | 38 | | 38 | | 38 | |
| *p* | 0.8241 | | 0.1579 | | 0.2202 | |
| Intercept |  |  |  |  |  |  |
| *t* | -1.3999 | | -2.2579 | | 0.1897 | |
| df | 39 | | 39 | | 39 | |
| *p* | 0.1694 | | 0.0296 | | 0.8505 | |
|  |  |  |  |  |  |  |
| Genotype | Napier | Mulato II | Mulato | Napier | Napier | Mulato |
| Treatment | Well-watered | Drought | Well-watered | Drought | Drought | Drought |
| n | 21 | 21 | 21 | 21 | 21 | 21 |
| *r* | 0.9738 | 0.953 | 0.972 | 0.86 | 0.86 | 0.953 |
| Slope |  |  |  |  |  |  |
| *t* | 0.3797 | | -0.5034 | | 0.4376 | |
| df | 38 | | 38 | | 38 | |
| *p* | 0.7063 | | 0.6176 | | 0.6642 | |
| Intercept |  |  |  |  |  |  |
| *t* | -3.0163 | | 1.3053 | | -1.2372 | |
| df | 39 | | 39 | | 39 | |
| *p* | 0.045 | | 0.1994 | | 0.2234 | |
